# A simple clinical model to predict presence of myocardial fibrosis in patients with aortic stenosis

**DOI:** 10.1186/1532-429X-18-S1-P235

**Published:** 2016-01-27

**Authors:** Vassilis Vassiliou, Tamir Malley, Simon Newsome, Calvin W Chin, Marc R Dweck, Dudley J Pennell, Sanjay Prasad

**Affiliations:** 1 grid.439338.6CMR, Royal Brompton Hospital, London, United Kingdom; 2 grid.8991.9000000040425469XLondon School of Hygiene and Tropical Medicine, London, United Kingdom; 3 grid.4305.20000000419367988Edinburgh University, Edinburgh, United Kingdom; 4 grid.7445.20000000121138111National Heart and Lung Institute, Imperial College London, London, United Kingdom

## Background

Both midwall and infarction-related fibrosis affect prognosis in patients with aortic stenosis. CMR remains the gold standard for identification of replacement fibrosis, however its use can be limited by patient suitability, cost and availability. We sought to develop a clinical model to identify the presence of fibrosis (midwall or infarction) based on variables from patient demographics, biomarker and imaging parameters which could potentially be applied easily in the outpatient setting.

## Methods

113 patients with moderate or severe aortic stenosis (age 78 [70, 83]; 71% males, average peak aortic valve gradient 59 mmHg) underwent transthoracic echocardiography to assess the severity of stenosis and CMR to determine the presence of midwall fibrosis or infarction. Forward stepwise selection was used to find the optimal logistic regression model to predict fibrosis from 91 potential predictors. This multivariable model was used to assign predicted probabilities of fibrosis to each patient, and checked using the Hosmer-Lemeshow goodness-of-fit test and the AUC statistic. The model was finally internally validated using a bootstrap method.

## Results

**Figure 1:**
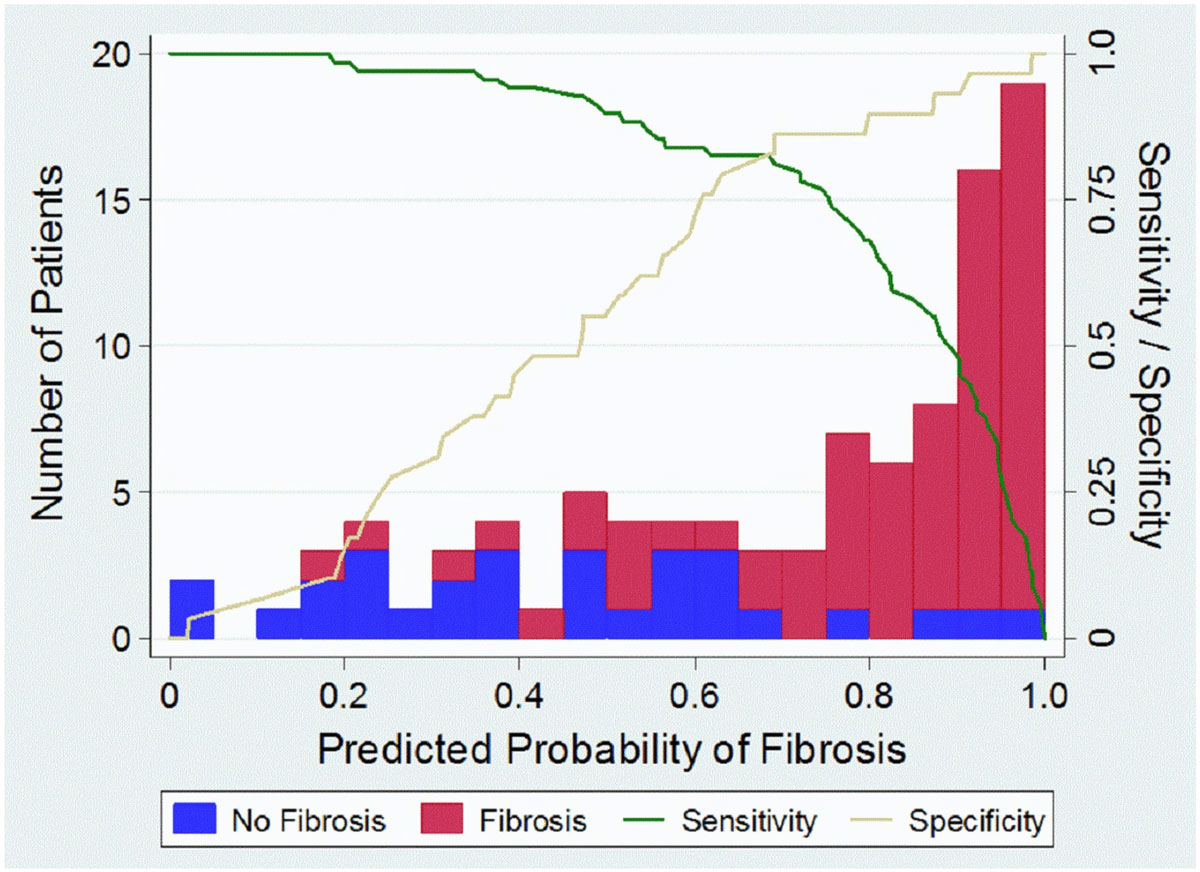
**Using the model based on the platelet count, urea level, NT-Pro BNP level and LVEF the probability of any fibrosis can be calculated for each patient**. Depending on the clinical setting the model allows for variable cut-off values to adjust sensitivity and specificity according to clinical requirements. For example, a probability cut-off of 0.50 will give 90% sensitivity of predicting any fibrosis with 55% specificity; whereas a cut-off value of 0.90 will give 50% sensitivity with a specificity of 93%.

In a multivariable model, platelet count, urea, LVEF (based on CMR) and NT-ProBNP remained significant. The final multivariable model was used to develop a clinical prediction model to assign a predicted probability of fibrosis to each patient. This was based on the three biomarkers and the LVEF. A histogram of these probabilities can be seen in figure 1, divided by whether the patients did or did not actually have fibrosis. Depending on the clinical setting, the information required and the probability cut-off chosen, the model can have sensitivity or specificity exceeding 90%.

A Hosmer-Lemeshow goodness-of-fit test was used to check the calibration of the model and there was no evidence that the model was badly calibrated (p=0.44). The AUC was 0.86, suggesting good discrimination. A bootstrap method was used to internally validate the discrimination of the model and the bias was estimated to be 3.9% (95% CI 0.2% -9.9%) suggesting only a small amount of bias.

## Conclusions

A clinical model to predict the presence or absence of fibrosis using simple clinical parameters is possible and can be used to risk-stratify patients easily in the outpatient setting.

